# A case of *Mycobacterium avium*-associated hypersensitivity pneumonitis

**DOI:** 10.1016/j.idcr.2025.e02464

**Published:** 2025-12-23

**Authors:** Akinari Atsumi, Takahiro Asami, Takuya Ozawa, Takanori Asakura, Ho Namkoong, Takashi Inoue

**Affiliations:** a Division of Pulmonary Medicine, Department of Medicine, Keio University School of Medicine, Tokyo, Japan; b Department of Internal Medicine, Sano Kosei General Hospital, Tochigi, Japan; c Department of Infectious Diseases, Keio University School of Medicine, Tokyo, Japan

**Keywords:** *Mycobacterium avium*, Hypersensitivity pneumonitis, Hot tub lung, NTM-PD

## Abstract

**Background:**

Hypersensitivity pneumonitis (HP) due to nontuberculous mycobacteria (NTM) is an uncommon phenotype of NTM pulmonary disease, classically linked to hot tub or pool exposure. We report a steroid-dependent case of HP-type *Mycobacterium avium* lung disease likely triggered by outdoor water aerosol exposure.

**Case presentation:**

A 79-year-old man with a 16-pack-year smoking history presented with progressive dyspnea on exertion. High-resolution CT showed bilateral peripheral ground-glass opacities, and serum Krebs von den Lungen-6 was markedly elevated. Bronchoalveolar lavage fluid demonstrated lymphocytic predominance with an increased CD4/CD8 ratio, fulfilling criteria for probable HP. Prednisolone induced clinical and radiological improvement; however, repeated attempts at tapering resulted in relapse with new ground-glass opacities in the right middle lobe and rising biomarkers. Although *Mycobacterium avium* had been isolated from bronchoalveolar lavage culture, the initial absence of respiratory symptoms and radiographic improvement led to observation alone. Given the difficulty tapering corticosteroids and the positive culture, chronic antigen exposure to environmental NTM was suspected. Further environmental assessment identified a long-standing habit of golfing on a riverside course, where ongoing inhalation of water aerosols was deemed the most likely source of antigenic exposure.

**Conclusion:**

Antigen avoidance combined with azithromycin and ethambutol led to sustained clinical and radiological improvement and successful steroid tapering without restrictive ventilatory impairment. This case underscores the importance of routinely sending mycobacterial cultures from bronchoalveolar lavage in suspected HP and of carefully reassessing environmental exposures, even in outdoor settings, as identifying NTM as the causative antigen can substantially modify management.

## Highlights

• Hypersensitivity pneumonitis triggered by Mycobacterium avium exposure.
• Recurrent ground-glass opacities during steroid taper raised suspicion of NTM.
• Antigen avoidance plus azithromycin–ethambutol allowed successful steroid reduction.
• BALF mycobacterial culture was crucial to identify NTM as the causative antigen.

## Description

**Fig. 1:**
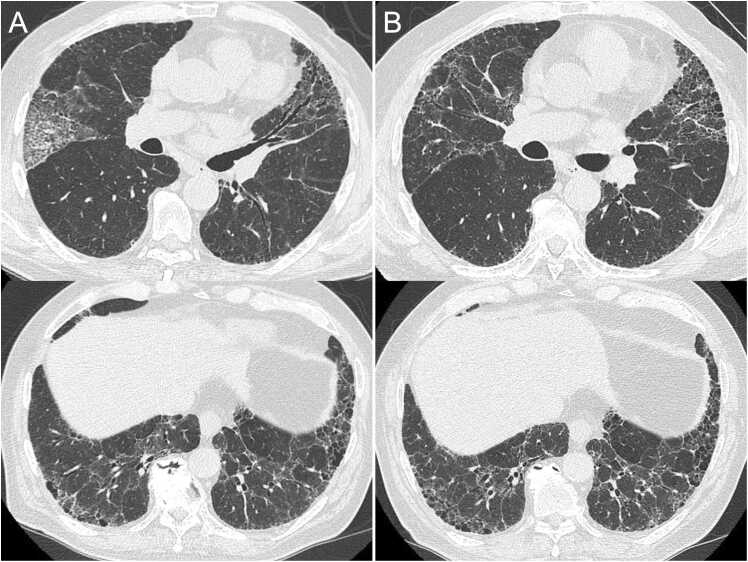
High-resolution computed tomography (HRCT) images. (A) Bilateral peripheral ground-glass opacities with a characteristic three-density pattern, obtained during relapse while tapering corticosteroid therapy. (B) One year after antigen avoidance and antimycobacterial therapy, the patient showed improvement in the middle lobe opacity.

A 79-year-old man with no relevant medical history presented with progressive dyspnea on exertion (DOE). He had a 16-pack-year smoking history and played golf near a riverside three times weekly. High-resolution computed tomography (HRCT) revealed peripheral, bilateral ground-glass opacities. Serum Krebs von den Lungen-6 (KL-6) was elevated at 1684 U/mL (reference range, <500 U/mL), and other laboratory findings, including connective-tissue disease autoantibodies were unremarkable. Bronchoalveolar lavage fluid (BALF) analysis revealed lymphocytic predominance (77 %) and increased CD4/CD8 ratio (7.40). The antigen exposure could not be identified, and the case was classified as probable hypersensitivity pneumonitis (HP). Prednisolone (PSL) (30 mg/day) administration improved symptoms and radiographic findings and decreased KL-6. The PSL dose was gradually tapered off. Although *Mycobacterium avium* was isolated from the BALF culture, the patient had no sputum production and showed radiologic improvement; therefore, we initially opted for careful observation. Four months post-treatment (PSL, 10 mg/day), DOE recurred, and KL-6 increased from 800 to 1246 U/mL, with new ground-glass opacities in the right middle lobe (Fig. 1**A**). PSL escalation to 15 mg/day again improved infiltrates and DOE, but relapse occurred when the dose was reduced to 12.5 mg/day. Because tapering PSL was difficult and BALF cultures were positive for mycobacteria, ongoing environmental exposure to nontuberculous mycobacteria (NTM) was suspected to underlie the persistent inflammation. Because he regularly played golf near the riverside, repeated aerosol exposure was considered the most plausible antigen source. Antigen avoidance therapy and combination therapy with azithromycin (250 mg/day) and ethambutol (500 mg/day) were initiated. This treatment improved symptoms and HRCT findings, allowing the PSL dose to be tapered to 5 mg/day (Fig. 1**B**). Furthermore, Forced Vital Capacity (FVC) remained preserved without evidence of restrictive ventilatory impairment.

HP-type NTM-PD represents a rare clinical phenotype typically linked to hot tubs, indoor pools, spas, and domestic showers [1], [2], [3], [4]. Although our patient had no such exposures, his frequent golfing near the riverside was considered the likely antigen source. BALF findings in HP-type NTM-PD typically demonstrate lymphocytic predominance with an elevated CD4/CD8 ratio [1]. Antigen avoidance is central to management. When this is inadequate, corticosteroid or antimycobacterial therapy may be considered, and rare cases require both [2]. The addition of antimycobacterial therapy in our patient improved disease control and facilitated corticosteroid tapering. This case highlights the importance of submitting BALF mycobacterial cultures for suspected HP, as the identification of NTM as an antigen source can significantly alter management.

Written informed consent was obtained from the patient for publication of this case report and the accompanying images.

## Funding sources

This work was supported by 10.13039/100009619AMED (JP25tm0424232) and JSPS KAKENHI (24KK0158, 25K02695).

## CRediT authorship contribution statement

**Akinari Atsumi:** Data curation, Investigation, Project administration, Writing – original draft. **Takahiro Asami:** Writing – review & editing. **Takanori Asakura:** Writing – review & editing. **Takuya Ozawa:** Writing – review & editing. **Takashi Inoue:** Supervision. **Ho Namkoong:** Supervision.

## Declaration of Competing Interest

The authors declare that they have no known competing financial interests or personal relationships that could have appeared to influence the work reported in this paper.

## Glossary

NTM: nontuberculous mycobacteria
HP: hypersensitivity pneumonitis
BALF: bronchoalveolar lavage fluid
HRCT: high-resolution computed tomography
GGO: ground-glass opacity
